# Knowledge, Attitude, and Practice Regarding Antibiotic Prescription Among Dentists in Saudi Arabia—A Cross-Sectional Survey

**DOI:** 10.3390/healthcare13101198

**Published:** 2025-05-20

**Authors:** Kiran Iyer, Jawaher Alhababi, Alanoud Aldhafayan, Amal Albarrak, Faey Alburidy, Fatimah Alanazi, Fatimah Albawardi, Abdulmohsen Alfadley

**Affiliations:** 1 Department of Preventive Dental Sciences, College of Dentistry, King Saud bin Abdulaziz University for Health Sciences, Riyadh 11426, Saudi Arabia; 2 King Abdullah International Medical Research Centre, Ministry of National Guard Health Affairs, Riyadh 11481, Saudi Arabia; fadleya@ksau-hs.edu.sa; 3 College of Dentistry, King Saud bin Abdulaziz University for Health Sciences, Riyadh 14611, Saudi Arabiaamalalbarrak0@gmail.com (A.A.); fay_elf@hotmail.com (F.A.); fatima.zaidan1999@gmail.com (F.A.); fatimah.abdulmalik00@gmail.com (F.A.); 4 Department of Restorative and Prosthetic Dental Sciences, College of Dentistry, King Saud bin Abdulaziz University for Health Sciences, Riyadh 11426, Saudi Arabia

**Keywords:** dentist, antibiotic, dental practitioner, analgesics, antimicrobial drug resistance, Saudi Arabia

## Abstract

**Background/Objectives:** Prescription challenges in antibiotics contribute to the global prevalence of antimicrobial resistance (AMR). We are rapidly moving into an age of ‘resistome’, which has grave consequences. Dentists frequently prescribe antibiotics for intraoral infections and as prophylaxis, particularly for immunocompromised patients, although the clinical justification for these prescriptions remains a point of concern. This study aimed to evaluate the knowledge, attitudes, and practices of dental practitioners in Saudi Arabia regarding antibiotic prescription. **Methods:** A cross-sectional survey was conducted from October to November 2024, involving 252 dentists from various regions of Saudi Arabia. Data were gathered through social media. **Results:** The dentists were likely to agree to prescribe antibiotics post-procedurally to be safe [OR 0.18, *p* = 0.42, CI −0.94]. Contrastingly, the knowledge of less experienced dentists [OR 9.60, *p* = 0.03, CI 1.21–76.15] was updated regarding prescribing penicillin for women in the third trimester of pregnancy than that of experienced dentists (>10 years). **Conclusions:** Although the knowledge level of practitioners in the public sector is reasonably good, there are concerns about antibiotic prescription practice among private dental practitioners.

## 1. Introduction

Antibiotic prescription in dental practice makes a small but significant contribution to the amount of antibiotics prescribed worldwide [1,2,3,4,5]. Dentists prescribe antibiotics to treat acute/chronic intraoral bacterial infections, while antibiotic prophylaxis is considered before dental treatment for individuals in an immunocompromised state. Other medical professionals such as internists, family practitioners, cardiologists, surgeons, pediatricians, and others may also prescribe and advise prophylactic antibiotics for dental infections and procedures. This suggests that antibiotic prophylaxis plays a significant role in the trending antibiotic prescription rates and their use when clinically indicated [6]. However, there is alarming concern that prophylactic antibiotics prescribed by dentists and medical fraternitymay contribute to the overall concern of antimicrobial resistance (AMR) [7].

Today, the concept of AMR is quickly moving to the concept of ‘resistome’. The World Health Organization (WHO) has proposed various clinical guidelines for eliminating antibiotic prescription overuse [8].

According to the American Association of Endodontists (AAE), antibiotic prophylaxis in dentistry is crucial to prevent infective endocarditis (IE) and implant joint infection in immunocompromised patients [9]. The American Heart Association (AHA) has also developed revised guidelines for antibiotic use for prophylaxis based on scientific evidence. The guidelines have been accepted and published by the American Dental Association (ADA) [10].

Recent studies investigating antibiotic prescription trends for both prophylaxis and treatment showed high rates of clinically inappropriate use of antibiotics. A retrospective cohort study analyzed the prophylactic use of antibiotics among dentists in the U.S. by collecting insurance data, which reported that 80% of the prescriptions were unjustified clinically [11]. A recent nationwide study by Rodríguez-Fernández A et al., among dentists in Spain, reported that more than half (59%) of their sample of 878 dentists gave out inappropriate antibiotic prescriptions for given case scenarios; hence, dentists exhibited a concerning trend of overusing antibiotics when it was not clinically indicated [12]. Overprescribing antibiotics affects the ecosystem at large. Astonishing data from the Centers for Disease Control and Prevention (CDC) reveal an annual mortality of 23,000 individuals in the United States because of AMR [7].

Kirchner et al.′s study in England revealed amoxicillin as the prescription of choice in 65% of overall antimicrobials prescribed by dentists. The study also revealed that the number of amoxicillin-resistant *E. coli* bacteria increased after using amoxicillin, potentially indicating the development of resistance [13]. Today, health professionals agree that the overall prescribing of antibiotics should be reduced and reserved for life-threatening infections to minimize the emergence of bacterial resistance to available antibiotics [14]. Given the role dentists play in the high rates of antibiotic prescriptions, efforts should be made to identify the causes of this issue.

The source of knowledge about antibiotic prescription for dental graduates in Saudi Arabia is primarily from their academic curriculum, which covers antimicrobial guidelines prepared by the antimicrobial stewardship technical subcommittee under the National Antimicrobial Resistance committee (AMR) [15]. The availability of over-the-counter antibiotics was checked by the Ministry of Health by introducing a law that resulted in a significant drop in the percentage of the availability of antibiotics without a prescription [16]. Previous studies on antimicrobial prescription patterns, dentists′ and general practitioners′ knowledge, and practice have been restricted to specific regions of Saudi Arabia. None of these studies tried to analyze the knowledge and practice regarding antimicrobial prescription based on the health status of patients undergoing dental treatment [17].

The present study was undertaken to address the above lacunae. Hence, it included a nationwide survey of dental practitioners through social media platforms to obtain an in-depth perspective on knowledge, practice, and attitude regarding antimicrobial prescription in this region.

## 2. Materials and Methods

The study methodology was based on STROBE guidelines for cross-sectional studies.

### 2.1. Study Design

The present study is cross-sectional and analytical in design, assessing the knowledge, attitude, and practice of dental practitioners in Saudi Arabia regarding antibiotic prescription.

### 2.2. Study Setting

This study was conducted from October 2024 to November 2024 (2 months), and a self-administered questionnaire was distributed through the social media platforms Telegram and X (previously known as Twitter). Ethical approval was obtained from the Institutional Review Board (NRR/24/022/9) of King Abdullah International Medical Research Center (KAIMRC), Riyadh, KSA, to ensure adherence to ethical standards.

### 2.3. Participants

A total of 252 dentists actively practicing in various dental specialties participated in this study. This study adopted a convenient sampling technique since samples were recruited through social media. The participants were drawn from diverse regions across Saudi Arabia, ensuring that the findings reflected a broader perspective with regard to knowledge, attitude, and practices within the dental community across the country. An estimated sample size of 240 was obtained based on correlations (r = 0.20) from previous research [17], with the confidence interval being 95% and power of study being 90%. Sample size estimation was performed using G power software (version 3.1.9.4). Informed consent was obtained from the participants before they could respond further to the questionnaire.

### 2.4. Questionnaire

The questionnaire was partially based on previous research [17,18], which allowed for previously validated questions, ensuring the assessment had a solid foundation in relevant scientific research. The previous research employed a questionnaire with 21 questions (eight demographic, eight practice-related, antibiotic prescription, and five clinical scenarios). We divided our questionnaire (knowledge and practice component) into topics regarding patients with and without systemic disease, and the present study adopted five questions with modifications.

This questionnaire consisted of four distinct sections. Much of the questionnaire consisted of close-ended questions, with only two open-ended questions to allow for free responses such as age and years of experience.

The initial section of the questionnaire intended to collect the participants′ demographic data (exploratory variables) such as gender, age, specialty, bachelor′s degree, years of experience, and region of practice in Saudi Arabia. This section also aimed to gain insight into the participants′ professional background.

The second section included an assessment of knowledge about antibiotic prescription, designed to evaluate understanding and clinical decision-making regarding antibiotic prescriptions. This section was divided into two main parts: one focusing on prescription knowledge for healthy patients and the second on prescription knowledge for patients with systemic disease. The response to knowledge questions was based on a 3-point scale (yes/no/not sure) and the type of drug administered.

The third section primarily focused on the practice aspect of prescribing antibiotics, specifically addressing scenarios, as in the knowledge component, for healthy patients and patients with specific medical diseases.

The fourth and final component of the questionnaire assessed dentists′ attitudes toward antibiotic prescribing practice, using a 5-point Likert scale from “strongly agree” to “strongly disagree”. This section presented seven attitude-related statements with scenarios, allowing respondents to express their agreement or disagreement with each statement. Responses to KAP-based questions were treated as dependent variables.

The questionnaire′s validity was carried out using Lawshe′s technique (1978) [19], with input from 5 subject experts on the importance of the questions and responses. When assessed for reliability on a pilot sample of 12 (5%) dentists [advanced education in general dentistry (AEGD) (4), endodontists (3), family dentists (3), and periodontists (2)] from the premises of the institution, the questionnaire elicited a Cronbach’s alpha value of 0.77, which is considered optimal. The participants in the pilot survey were not part of the final analysis.

### 2.5. Statistical Analysis

The data were transferred to Microsoft Excel (Microsoft Corp., New York, NY, USA) initially for data cleaning and coding and were later transferred into the Statistical Package for the Social Sciences (SPSS) statistical software (VERSION 20, 2011; IBM Corp., Armonk, NY, USA) for analysis; descriptive statistics were used to describe the demographic data based on frequency and percentage. Fisher′s exact test was used to cross-tabulate the categorical variables and ascertain the significance. As a second step, multinomial logistic regression was applied to assess the significance of the exploratory variables found significant during the initial analysis from Fischer′s exact test. Adjusted odds were reported after adjusting for covariates such as years of experience and region of practice.

## 3. Results

A total of 252 dentists responded to the questionnaire, of whom 130 (51.6%) and 121 (48.4%) were female and male respondents, respectively. The mean age of the dentists was 31.5 (±6.4) years, and their mean professional experience was 5.6 (±5) years. Based on the data, general practitioners comprised 172 (68.3%) dentists who responded to the questionnaire. Conversely, oral radiology 01 (0.4%) was the least represented specialty. Other specialties represented were periodontics—9 (3.6%); pediatric dentistry—11 (4.4%); orthodontics—6 (2.4%); and prosthodontics—18 (7.1%), among others.

Of most dentists, 247 (98%) obtained their bachelor’s degree from countries in the Gulf Cooperation Council (GCC) region, and most of these dentists, 118 (46.8%), practiced in the central geographic area. About 114 (46.6%) of the dentists practiced in public (academic/hospital setting), 48 (19.1%) in private (academic/hospital setting), and the remaining 90 (35.7%) had private practices (Table 1).

Knowledge about antibiotic prescription among dentists for healthy individuals ascertained a significant difference in four of the five knowledge questions. The type of dental setting practice (*p* = 0.001) and specialty (*p* = 0.001) significantly influenced the response to the question “*Is penicillin administered to a pregnant woman if it is required in the third trimester?*”

Those from the public practice setup (academic/hospital) responded appropriately. Specialists in pediatric dentistry and general dentists responded that they were unsure of antibiotic prescriptions in the third trimester of pregnancy (Table 2).

Interestingly, the region of practice (*p* = 0.00) and where one acquired their degree from (*p* = 0.004) also significantly varied in response to “*Is antibiotic prescribed for visible sinus tract on attached gingiva?*”. Dentists from the central region and those who graduated from other countries positively affirmed a need for antibiotic prescription. Likewise, graduates from other countries were significantly (*p* = 0.034) more likely to prescribe an antibiotic in cases of surgically impacted third molars and reversible pulpitis (*p* = 0.022) (Table 2).

The summation of variables within the data led to an increased degree of freedom (df), as observed in the results.

Knowledge of dentists about antibiotic prescription after extraction in patients with uncontrolled diabetes elicited a significant (*p* = 0.001) variation in response based on the dentist’s workplace setting; those in the public sector (academic/hospital) were more likely to not prescribe a drug than dentists in a private dental setup. Similarly, general dentists were significantly (*p* = 0.000) unlikely to prescribe antibiotics in similar conditions compared to other dental specialists. No significance (*p* = 0.359) was observed considering the experience of dentists and their prescription patterns of antibiotics for patients with a history of IE undergoing extraction (Table 3).

Like knowledge-related questions, practice questions were divided into subcategories of antibiotic prescription in patients with and without systemic disease. A significantly (*p* = 0.001) high percentage (72%) of dentists in private (academic/hospital) setups responded with the practice of prescribing clindamycin. About 46 (18%) respondents were misled with the option of augmentin, a combination of amoxicillin and clavulanic acid. Clindamycin was a significant (*p* = 0.011) alternative drug of choice to amoxicillin among the practitioners in the country′s central region (Table 4).

General dentists clearly and significantly (*p* = 0.031) indicated that they did not prescribe drugs after simple extraction compared to other specialty dentists (Table 4).

A significant (*p* = 0.031) number of public (academic/hospital) dental practitioners, 97 (83%), mentioned the use of amoxicillin for patients with a history of heart valve replacement in the prior year. Amoxicillin was significantly the drug of choice for dentists irrespective of the region (*p* = 0.024) or experience (*p* = 0.001), compared to higher broad-spectrum antibiotics such as augmentin or clindamycin (Table 5).

Prescribing no drug over antibiotics was significantly the choice of dental practitioners for patients after gingivectomy and for those with a medical history of hypertension, irrespective of region (*p* = 0.032) or years of experience (*p* = 0.001) (Table 5).

Dental practitioners had a significant (*p* = 0.003) and favorable attitude, agreeing with a need for antibiotic prophylaxis in patients with a relevant medical history. However, through Fisher′s exact test, it could not be indeed established whether private (academic/hospital) dental practitioners disagreed with antibiotic prophylaxis for the same (Table 6).

Contradicting the usual antibiotic use norms and with a focus on antibiotic resistance, it was interesting to note that a significantly high number of dental practitioners in the experience bracket of less than 10 years felt the need to prescribe antibiotics post-procedurally to be safe (Table 6).

Responses to questions were considered the dependent variable assessed against independent demographic variables of the participating dentists. An unadjusted odds ratio was initially assessed for each of the independent variables; the outcome variable was adjusted with the years of experience of the practitioners and region of practice as covariates for knowledge-, practice-, and attitude-based questions. Dental practitioners with less than 10 years, 1 to 5 years (OR 2.26, *p* = 0.03, CI 1.21–76.15), and 6–10 years (OR 3.32, *p* = 0.02, CI−3.4–84.5) of experience had a better knowledge of administrating penicillin in the third trimester of pregnancy compared to their fellow dentists with more than 10 years of experience. Dentists practicing in the central region (OR−0.50, *p* = 0.28, CI 0.23–1.53) were less likely to prescribe antibiotics after a simple extraction compared to practitioners from other regions of the country, whereas interesting years of experience in respective regions seem to significantly influence the prescribing pattern in all regions, as observed after adjusting for the experience. Contrasting with their knowledge response, dentists with less than 10 years (OR 0.55, *p* = 0.46, CI 0.39–7.62) of experience were more likely to prescribe antibiotics post procedurally than practitioners with more than 10 years of experience; the response does not vary after being adjusted for the region of practice (Table 7).

## 4. Discussion

The present study received 252 responses from dentists across different regions in Saudi Arabia, so a broad national representation was achieved within the sample. Inappropriate antibiotic use has been considered a public health concern and a contributing factor to the rise in antimicrobial resistance (AMR) [7,8].

Although there is similar research among dentists in the KSA, the present study attempted to address the shortcomings of previous studies, such as low sample size and studies that did not assess the practice based on dental setting [18]. In certain similar studies, students and academicians were considered for the final sample; this study comprehensively considered all the factors and addressed the above lacunae [20]. This study included practitioners associated with the public sector (hospitals/colleges), those associated with the private sector (hospitals/colleges), and private practitioners. This study also assessed the knowledge and practice of these practitioners regarding antibiotic usage among healthy patients and patients with compromised systemic health/health issues undergoing dental treatment.

The present study revealed significant uncertainty among practitioners, particularly general dentists, regarding the use of antibiotics like penicillin during pregnancy, especially in the third trimester, for healthy individuals. Meanwhile, public sector practitioners had better antibiotic prescription knowledge than private practitioners about penicillin administration during pregnancy. Our study also revealed uncertainty among pediatric dental specialists and inadequate knowledge among general dentists regarding penicillin use during the third trimester of pregnancy. To address this, the American Dental Association Council on Scientific Affairs recommends advising patients on antibiotic interactions with oral contraceptives, suggesting nonhormonal alternatives, and ensuring compliance. While penicillin is generally safe during pregnancy, tetracyclines are contraindicated due to the risk of dental discoloration [21].

In our study, the majority responded “No” to prescribing antibiotics for healthy individuals with visible sinus tracts, aligning with findings from a study conducted in Riyadh by Baskaradoss et al. [22]. Conversely, a study evaluating dental students′ knowledge and attitudes towards antibiotics by AboAlsamh et al. revealed that most participants favored prescribing antibiotics for their patients [23].

The prescription of antibiotics is not indicated for the surgical extraction of impacted third molars unless there is systemic involvement or a preexisting local infection, as noted in the guidelines [24,25]. The frequency of dentists refraining from prescribing antibiotics after the surgical extraction of a third molar is significantly higher compared to findings from the study by Baskaradoss et al. [22]. This study examined the antibiotic prescription pattern among dentists in Riyadh, Saudi Arabia, recommending stringent guidelines to minimize unnecessary antibiotic use. Their findings support the principle that antibiotics should not be routinely prescribed as a preventive measure for third-molar extractions. Instead, they are reserved for specific cases involving high-risk patients or severe infections, ensuring a more targeted and evidence-based approach to antibiotic use [21].

Based on the findings from our study, a significant number of participants answered ‘no’ to the prescription of antibiotics for reversible pulpitis in healthy individuals, in comparison to the study by AboAlSamh et al. [23], which examined dental students’ knowledge and attitudes towards antibiotic prescribing guidelines in Riyadh, Saudi Arabia. Their study highlighted discrepancies in antibiotic prescription practices among dental students, with many indicating uncertainty about when antibiotics should be prescribed [19].

Our findings show that dentists, irrespective of dental setting and specialty, prefer prescribing amoxicillin as a drug of choice in patients with uncontrolled diabetes and in those who have undergone invasive dental procedures such as the extraction of impacted third molars, which is in line with the guidelines given in the study by Oberoi et al. [26]. Amoxicillin was also the drug of choice for dentists when their knowledge was assessed for patients with a history of infective endocarditis undergoing simple extraction; this makes evident the fact that the knowledge was fairly good among the responding dentists in our study [8,9].

Amoxicillin is the most prescribed antibiotic for dental treatments worldwide [1], including for individuals with systemic disorders, while clindamycin is used as an alternative for patients with confirmed penicillin allergies [27]. Similarly, a high frequency of dentists claimed to prescribe clindamycin as an alternative AB in the present study, with one of its significant drawbacks being the risk of inducing Clostridioides difficile infections. A 2020 study from Colombia revealed that more than half of the surveyed dentists preferred amoxicillin as a first-line antibiotic, with clindamycin being the second choice [28].

Our study found that most general dentists do not prescribe antibiotics for healthy patients undergoing simple extractions, which aligns with the Oral and Maxillofacial Surgery guidelines. These guidelines suggest that antibiotics are unnecessary unless there is a risk factor for infective endocarditis (IE) [25]. Amoxicillin was the drug of choice among practicing dentists, regardless of dental practice setting, region of practice, or years of experience, for patients who underwent valve replacement a year prior [1,2,3,4,5,20]. Dentists unanimously responded that ‘no’ antibiotic should be prescribed for patients with hypertension undergoing gingivectomy, as this aligns with current guidelines emphasizing that antibiotic prophylaxis is unnecessary unless systemic risk factors or active infections are present [29]. The dentists in the public sector were more likely to ask the patients about their medical condition to avoid postoperative IE compared to the dentists in the private sector. A study from Jeddah also reported similar results [30]. Additionally, research from Kuwait showed that dentists with more professional experience had a better understanding of how antibiotics are appropriately used, which aligns with our findings [31]. Less experienced dentists (<10 years) are more likely to prescribe antibiotics post-procedurally to ‘be safe,’ raising concerns about antibiotic overuse and antimicrobial resistance [28].

## 5. Conclusions

This study highlights variations in knowledge, attitude, and practice regarding antibiotic prescription among dental practitioners working in various sectors in Saudi Arabia. Dentists practicing in the public sector (academic/hospital) had better knowledge compared to their private-sector counterparts. Younger dentists exhibited better knowledge about prescription, which may be due to updated knowledge as part of their having recently graduated or because of the continuing professional dental education being received by this group. Interestingly, senior practitioners (<10 years of experience) exhibited more significant knowledge in the practice questions.

This study′s main limitation was the insufficient sample size (responses) from specialized dentists and dentists who graduated from other regions of the world and are presently practicing in Saudi Arabia. The need for a higher sample size is reflected through the large confidence interval for certain responses in this study. Future research should be undertaken ensuring that large samples are recruited over a longer duration, allowing for more responses, or approaching dentists through regional councils in order to ensure easier follow-up and reminders for dentists, which would help overcome the lacunae of the present study. The low sample size necessitated the use of Fisher’s exact test for analysis. Construct validity of the questionnaire could be undertaken to evaluate the underlying contribution of questions and drop the laggards; item total and item deletion statistics could be undertaken towards this end. Antibiotic awareness is at a crossroads and must be addressed among all dental practitioners throughout the country. The authors recommend that the regional council mandate that at least one of the continuing medical education lectures/workshops/seminars attended by dentists be related to antibiotic use and guidelines, ensuring better knowledge, attitude, and safe/conservative practice regarding antibiotic use.

## Figures and Tables

**Table 1:** Demographic details of the participating dentists.

Demographic Variable	Sub-Division	Total (N)	Mean	Standard Deviation (±)	Minimum	Maximum
**Demographic Variable**	-	Total	Mean	Standard Deviation (±)	Frequency (n)	Percentage (%)
Specialty of Dentists	Advanced Education in General Dentistry (AEGD)	252	-	-	09	4
	Dental Public Health				02	1
	Endodontist				06	2
	Family Dentistry				03	1
	General practice				172	68
	Oral Medicine				04	2
	Oral Radiology				01	0.4
	Oral Surgery				03	1
	Orthodontics				06	2
	Pediatric Dentistry				11	4
	Periodontics				09	4
	Prosthodontic				18	7
	Restorative Dentistry				08	3
Gender	Male	252	-	-	122	48
	Female				130	52
Dentist with degree obtained from	Africa	252	-	-	01	0.4
	Asia				02	1
	GCC countries (Saudi Arabia, Qatar, UAE, Kuwait, Bahrain, Oman)				247	98
	North America				02	1
Region of Dental Practice	Central	252	-	-	118	47
	Eastern				42	17
	Northern				20	8
	Southern				39	16
	Western				30	13
Dental Practice Setting	Academic/Hospital/Public	252	-	-	114	55
	Academic/Hospital/Private				48	19
	Private Practice				90	36

Most percentages are rounded to the nearest whole number.

**Table 2:** Knowledge of dentists about antibiotic prescription in healthy individuals.

Q. Is Penicillin Administered to Pregnant Women If It Is Required in the Third Trimester?							
	Dental Setting	*n* (%)			Total	df	Fisher’s exact test (*p*-Value)
		Yes	No	Not Sure			
	Academic/Hospital/Private	27 (59)	7 (15)	12 (26)	46 (100)	15	0.001 *
	Academic/Hospital/Public	58 (49)	46 (40)	13 (11)	117 (100)		
	Private Practice	37 (42)	44 (49)	8 (9)	89 (100)		
	Total	122 (48)	97 (39)	33 (13)	252 (100)		
	**Specialty**	*n* (%)			Total	24	0.010 *
		Yes	No	Not Sure			
	Advanced Education in General Dentistry (AEGD)	6 (67)	2 (22)	1 (11)	9 (100)		
	Endodontics	5 (83)	0 (0)	1 (17)	6 (100)		
	Pediatric Dentistry	2 (18)	0 (0)	9 (82)	11 (100)		
	Periodontics	4 (45)	2 (22)	3 (33)	9 (100)		
	Prosthodontics	12 (66)	3 (17)	3 (17)	18 (100)		
	General Practitioner	66 (48)	82 (14)	24 (38)	172 (100)		
	All Other Specialty #	20 (74)	6 (22)	1 (4)	27 (100)		
	Total	115 (46)	95 (38)	42 (16)	252 (100)		
**Q. Is antibiotic prescribed for a patient with a visible sinus tract in the attached gingiva?**							
	**Region of Practice**	*n* (%)			Total	20	0.004 *
		Yes	No	Not Sure			
	Central	25 (21)	84 (71)	9 (8)	118 (100)		
	Eastern	5 (12)	34 (81)	3 (7)	42 (100)		
	Western	1 (3)	28 (85)	4 (12)	33 (100)		
	Northern	8 (40)	11 (55)	1 (5)	20 (100)		
	Southern	2 (5)	30 (77)	7 (18)	39 (100)		
	Total	41 (16)	187 (74)	24 (10)	252 (100)		
	**Degree based on country**	*n* (%)			Total	15	0.001 *
		Yes	No	Not Sure			
	GCC	36 (15)	187 (75)	24 (10)	247 (100)		
	Other	5 (2)	0 (0)	0 (0)	5 (100)		
	Total	41 (17)	187 (75)	24 (10)	252 (100)		
**Q. Would you prescribe antibiotics for a surgically impacted third molar?**							
	**Degree based on country**	*n* (%)			Total	2	0.034 *
		Yes	No	Not Sure			
	GCC	63 (25)	165 (67)	19 (8)	247 (100)		
	Other #	4 (80)	1 (20)	0 (0)	05 (100)		
	Total	67 (27)	166 (66)	19 (7)	252 (100)		
**Q. Would you prescribe antibiotics for reversible pulpitis?**							
	**Degree based on Country**	*n* (%)			Total	2	0.022 *
		Yes	No	Not Sure			
	GCC	9 (4)	232 (94)	6 (2)	247 (100)		
	Other	2 (40)	3 (60)	0 (0)	05 (100)		
	Total	11 (4)	235 (94)	6 (2)	252 (100)		

* Significance—*p* value (<0.05); Other—North America (02), Africa (01), and Asia (02); all other specialties # (dental public health, oral medicine, oral surgery, oral radiology, family dentistry, orthodontics, restorative); and certain df (degrees of freedom) are higher due to the variation in the sample and summation.

**Table 3:** Knowledge of dentists about antibiotic prescription for dental procedures in individuals with systemic disease.

Q. Which of the Following Drugs is Prescribed for a Patient with Uncontrolled Diabetes after Extraction of the Impacted Third Molar?									
	Dental Setting	*n* (%)					Total	df	Fisher’s exact test (*p*-Value)
		Amoxicillin	Augmentin	Clindamycin	Metronidazole	No Drug Prescribed			
	Academic/ Hospital/ Private	34 (74)	0 (0)	4 (9)	2 (4)	6 (13)	46 (100)	12	0.001 *
	Academic/ Hospital/ Public	63 (54)	1 (1)	5 (4)	33 (28)	15 (13)	117 (100)		
	Private Practice	49 (55)	1 (1)	4 (4)	31 (36)	4 (4)	89 (100)		
	Total	144 (58)	2 (1)	13 (5)	66 (26)	23 (10)	252 (100)		
	Specialty	*n* (%)					Total	72	0.000 *
		Amoxicillin	Metronidazole			No Drug Prescribed			
	AEGD	8 (89)	0 (0)			1 (11)	9 (100)		
	Endodontics	4 (67)	0 (0)			2 (33)	6 (100)		
	Periodontics	4 (44)	1 (11)			4 (44)	9 (100)		
	Prosthodontics	16 (89)	1 (5)			1 (5)	18 (100)		
	Pediatric Dentistry	7 (64)	0 (0)			4 (36)	11 (100)		
	General Practitioner	103 (60)	63 (37)			6 (3)	172 (100)		
	All Other Specialties #	18 (67)	2 (7)			7 (26)	27 (100)		
	Total	160 (63)	67 (27)			25 (10)	252 (100)		
**Q. Which of the following drugs is prescribed for a patient with a history of Infective Endocarditis after simple extraction?**									
	Years of Experience	*n* (%)					Total	df	Fisher’s exact test (*p*-Value)
		Amoxicillin	Clindamycin		Metronidazole	No Drug Prescribed			
	1 to 5	120 (87)	11 (8)		4 (3)	3 (2)	138 (100)	12	0.359
	6 to 10	78 (93)	1 (1)		1 (1)	3 (4)	83 (100)		
	11 to 15	13 (87)	1 (6)		0 (0)	1 (6)	15 (100)		
	16 to 22	14 (88)	0 (0)		0 (0)	2 (12)	16 (100)		
	Total	225 (89)	13 (5)		5 (2)	9 (4)	252 (100)		

* Significance—*p* value (<0.05), all other specialties # (dental public health, oral medicine, oral surgery, oral radiology, family dentistry, orthodontics, restorative), and certain df (degrees of freedom) are higher due to variation in the sample and summation.

**Table 4:** Dentist practice of antibiotic prescription for dental procedures in individuals with no medical history.

Q. What Alternative Antibiotic Do You Commonly Prescribe for Penicillin-Allergic Patients?								
	Dental Setting	*n* (%)			Total		df	Fisher’s exact test *p*-Value
		**Augmentin**	**Clindamycin**	**Tetracycline**				
	Academic/ Hospital/ Private	3 (7)	42 (91)	1 (2)	46 (100)		4	0.001 *
	Academic/ Hospital/ Public	25 (21)	75 (64)	17 (15)	117 (100)			
	Private Practice	18 (20)	64 (72)	7 (8)	89 (100)			
	Total	46 (18)	181 (72)	25 (10)	252 (100)			
	**Region of Practice**	*n* (%)			Total		8	0.011 *
		Augmentin	Clindamycin	Tetracycline				
	Central	13 (11)	96 (81)	9 (8)	118 (100)			
	Eastern	6 (14)	28 (67)	8 (19)	42 (100)			
	Western	11 (33)	18 (55)	4 (12)	33 (100)			
	Northen	7 (35)	12 (60)	1 (5)	20 (100)			
	Southern	9 (23)	27 (69)	3 (8)	39 (100)			
	Total	46 (18)	181 (72)	25 (10)	252 (100)			
**Q. What do you prescribe for a patient after a simple extraction?**								
	**Specialty**	*n* (%)				Total	36	0.031 *
		Amoxicillin	Analgesic	Clindamycin	No Drug Prescribed			
	AEGD	4 (44)	0 (0)	0 (0)	5 (56)	9 (100)		
	Endodontics	3 (50)	1 (17)	0 (0)	2 (33)	6 (100)		
	Periodontics	1 (11)	0 (0)	0 (0)	8 (89)	9 (100)		
	Prosthodontics	6 (33)	0 (0)	0 (0)	12 (67)	18 (100)		
	Pediatric Dentistry	1 (9)	2 (18)	0 (0)	8 (73)	11 (100)		
	General Practitioner	32 (19)	1 (1)	4 (2)	135 (79)	172 (100)		
	All Other Specialty #	10 (37)	0 (0)	0 (0)	17 (63)	27 (100)		
	Total	57 (23)	4 (2)	4 (2)	187 (73)	252 (100)		
	**Region of Practice**	*n* (%)				Total	12	0.030 *
		Amoxicillin	Analgesic Only	Clindamycin	No Drug Prescribed			
	Central	21 (18)	0 (0)	1 (1)	96 (81)	118 (100)		
	Eastern	12 (29)	0 (0)	0 (0)	30 (71)	42 (100)		
	Western	8 (24)	2 (6)	1 (3)	22 (67)	33 (100)		
	Northen	5 (25)	0 (0)	2 (10)	13 (65)	20 (100)		
	Southern	10 (26)	2 (5)	0 (0)	27 (69)	39 (100)		
	Total	56 (22)	4 (2)	4 (2)	188 (75)	252 (100)		
**Q. What antibiotic do you prescribe for a patient with chronic necrotic pulp?**								
	**Degree based on Country**	*n* (%)			Total		2	0.041 *
		Amoxicillin	Clindamycin	No Drug Prescribed				
	GCC	36 (15)	3 (1)	208 (84)	247 (100)			
	Other	2 (40)	1 (20)	2 (40)	05 (100)			
	Total	38 (15)	4 (2)	210 (83)	252 (100)			

* Significance—*p* value (<0.05) and certain df (degrees of freedom) are higher due to variation in sample and summation. # (dental public health, oral medicine, oral surgery, oral radiology, family dentistry, orthodontics, restorative).

**Table 5:** Dentist practice of antibiotic prescription for dental procedures for individuals with relevant medical history.

Q. What Antibiotic Would You Prescribe for a Patient with Valve Replacement 1 Year Ago?								
	Dental Setting	*n* (%)				Total	df	Fisher’s exact test *p*-Value
		Amoxicillin	Augmentin	Clindamycin	No Drug Prescribed			
	Academic/Hospital/ Private	30 (65)	0 (0)	4 (9)	12 (26)	46 (100)	6	0.031 *
	Academic/Hospital/ Public	97 (83)	2 (2)	1 (1)	17 (15)	117 (100)		
	Private Practice	75 (84)	0 (0)	3 (3)	11 (12)	89 (100)		
	Total	202 (80)	2 (1)	8 (3)	40 (16)	252 (100)		
	Region of Practice	*n* (%)				Total	12	0.024 *
		Amoxicillin	Augmentin	Clindamycin	No Drug Prescribed			
	Central	87 (74)	2 (1)	3 (3)	26 (22)	118 (100)		
	Eastern	38 (90)	0 (0)	1 (2)	3 (7)	42 (100)		
	Western	26 (79)	0 (0)	0 (0)	7 (21)	33 (100)		
	Northen	17 (85)	0 (0)	3 (15)	0 (0)	20 (100)		
	Southern	34 (87)	0 (0)	1 (3)	4 (10)	39 (100)		
	Total	202 (80)	2 (1)	8 (3)	40 (16)	252 (100)		
	Years of Experience	*n* (%)				Total	9	0.001 *
		Amoxicillin	Augmentin	Clindamycin	No Drug Prescribed			
	1 to 5	96 (70)	1 (1)	7 (5)	34 (25)	138 (100)		
	6 to 10	77 (93)	0 (0)	1 (1)	5 (6)	83 (100)		
	11 to 15	13 (87)	1 (6)	0 (0)	1 (6)	15 (100)		
	16 to 22	16 (100)	0 (0)	0 (0)	0 (0)	16 (100)		
	Total	202 (80)	2 (1)	8 (3)	40 (16)	252 (100)		
**Q. What antibiotic do you prescribe for patients with hypertension after gingivectomy?**								
	Region of Practice	*n* (%)				Total	df	Fisher’s exact test *p*-Value
		Amoxicillin	Clindamycin		No Drug Prescribed			
	Central	22 (19)	6 (5)		90 (76)	118 (100)	8	0.032 *
	Eastern	10 (24)	0 (0)		32 (76)	42 (100)		
	Western	3 (8)	1 (3)		29 (89)	33 (100)		
	Northen	9 (45)	0 (0)		11 (55)	20 (100)		
	Southern	5 (13)	4 (10)		30 (77)	39 (100)		
	Total	49 (19)	11 (4)		192 (76)	252 (100)		
	Years of Experience	Amoxicillin	Clindamycin		No Drug Prescribed	Total	6	0.001 *
	1 to 5	27 (20)	10 (7)		101 (73)	138 (100)		
	6 to 10	9 (11)	1 (1)		73 (88)	83 (100)		
	11 to 15	10 (67)	0 (0)		5 (33)	15 (100)		
	16 to 22	3 (18)	0 (0)		13 (81)	16 (100)		
	Total	41 (20)	11 (4)		192 (76)	252 (100)		

* Significance—*p* value (<0.05) and certain df (degrees of freedom ≥12) are higher due to variation in the sample and its summation.

**Table 6:** Dentists′ attitudes regarding antibiotic prescription for dental procedures.

Q. I Ask Patients If They Have Any Medical Conditions Typically Requiring Antimicrobial Prophylaxis Against IE Before Performing Dental Procedure?									
	**Dental Setting**	*n* (%)					Total	df	Fisher’s exact test (*p*-Value)
		Strongly Agree	Agree	Neutral	Disagree	Strongly Disagree			
	Academic/ Hospital/ Private	15 (33)	3 (7)	1 (2)	27 (58)	0 (0)	46 (100)	8	0.003 *
	Academic/ Hospital/ Public	60 (51)	0 (0)	10 (9)	47 (40)	0 (0)	117 (100)		
	Private Practice	50 (56)	0 (0)	7 (8)	31 (35)	1 (1)	89 (100)		
	Total	125 (49)	3 (1)	18 (7)	105 (42)	1 (1)	252 (100)		
**Q.** **I continue to prescribe antimicrobials post procedurally just to be safe**									
	**Specialty**	*n* (%)					Total	48	0.007 *
		Strongly Agree	Agree	Neutral	Disagree	Strongly Disagree			
	AEGD	0 (0)	1 (11)	1 (11)	4 (44)	3 (33)	9 (100)		
	Endodontics	2 (33)	1 (17)	0 (0)	1 (17)	2 (33)	6 (100)		
	Periodontics	0 (0)	3 (33)	1 (11)	1 (11)	4 (44)	9 (100)		
	Prosthodontics	0 (0)	3 (17)	4 (22)	10 (56)	1 (5)	18 (100)		
	Pediatric Dentistry	0 (0)	1 (9)	3 (27)	5 (46)	2 (18)	11 (100)		
	General Practitioner	21 (12)	55 (32)	28 (16)	31 (18)	37 (22)	172 (100)		
	All Other Specialty #	3 (11)	3 (30)	7 (26)	8 (11)	6 (22)	27 (100)		
	Total	26 (10)	67 (27)	44 (17)	60 (24)	55 (22)	252 (100)		
	**Years of Experience**	*n* (%)					Total	12	0.001 *
		Strongly Agree	Agree	Neutral	Disagree	Strongly Disagree			
	1 to 5	11 (8)	23 (17)	31 (12)	33 (22)	40 (29)	138 (100)		
	6 to 10	13 (16)	37 (45)	3 (4)	23 (27)	7 (8)	83 (100)		
	11 to 15	2 (13)	3 (20)	3 (20)	3 (20)	4 (27)	15 (100)		
	16 to 22	0 (0)	4 (25)	7 (44)	1 (6)	4 (25)	16 (100)		
	Total	26 (10)	67 (27)	44 (17)	60 (24)	55 (22)	252 (100)		

* Significance—*p* value (<0.05), all other specialties # (dental public health, oral medicine, oral surgery, oral radiology, family dentistry, orthodontics, restorative), and certain df (degrees of freedom ≥12) are higher due to variation in sample and its summation.

**Table 7:** Multinomial regression analysis of important dependent variables found to be significant with Fisher′s exact test.

Variable	Independent Variable	B	df	Sig.	Exp (B)	95% Confidence Interval for Exp (B)		Adjusted Exp (B)	Sig.	95% Confidence Interval for Adjusted Exp (B)	
						Lower Bound	Upper Bound			Lower Bound	Upper Bound
**Is penicillin administered to pregnant women if it is required in the third trimester?** **(Knowledge Component)**	Years of Experience	Response: No ^#,a^						Adjusted for Region of Practice			
	1 to 5	2.26	1	0.03 *	9.60	1.21	76.15	2.31	0.80	6.26	8.54
	6 to 10	3.32	1	0.02 *	27.8	3.4	84.5	2.43	0.92	6.59	9.02
	11 to 15	1.59	1	0.19	4.93	0.44	55.4	2.50	0.89	6.77	9.24
**What antibiotic would you prescribe for a patient who had valve replacement 1 year ago?** **(Knowledge Component)**	Dental Setting	Response: No Drug Prescribed ^#,b^						Adjusted for Years of Experience			
	Academic/ Hospital/ Private	1.00	1	0.03 *	2.72	1.08	6.85	0.02	0.08	0.01	0.75
	Academic/ Hospital/ Public	0.17	1	0.66	1.19	0.52	2.70	1.16	0.78	0.38	3.52
**What do you prescribe for a patient after simple extraction?** **(Practice Component)**	Region of Practice	Response: Amoxicillin ^#,c^						Adjusted for Years of Experience			
	Central	−0.50	1	0.28	0.60	0.23	1.53	4.15	0.00 *	4.98	5.26
	Eastern	0.09	1	0.85	1.10	0.38	3.14	1.68	0.00 *	11.21	25.46
	Northen	0.05	1	0.93	1.05	0.28	3.92	1.87	0.00 *	7.17	8.88
	Southern	0.01	1	0.97	1.01	0.34	3.02	1.78	0.00 *	2.12	2.26
**I continue to prescribe anti-microbials post-procedurally just to be safe** **(Attitude Component)**	Years of Experience	Response: Strongly Disagree ^#,d^						Adjusted for Region of Practice			
	1 to 5	0.55	1	0.46	1.73	0.39	7.62	0.70	0.92	0.61	1.38
	6 to 10	−1.66	1	0.42	0.18	0.38	0.94	0.78	0.93	0.58	1.49
	11 to 15	0.28	1	0.78	1.33	0.17	10.25	0.69	0.92	0.60	1.39

# Reference category: a—16–22 (last), b—private practice (last); c—no drug prescribed (last); d—strongly agree (first); and * significance—*p* value (<0.05).

## Data Availability

Data are available in a publicly accessible repository. The data presented in this study are openly available in [Fig Share] at https://doi.org/10.6084/m9.figshare.28035479.v1 (accessed on 16 December 2024).
